# piRNA pathway evolution beyond gonad context: Perspectives from apicomplexa and trypanosomatids

**DOI:** 10.3389/fgene.2023.1129194

**Published:** 2023-02-03

**Authors:** S. Horjales, M Li Calzi, M. E. Francia, A. Cayota, M. R. Garcia-Silva

**Affiliations:** ^1^ Apicomplexa Biology Laboratory, Institute Pasteur Montevideo, Montevideo, Uruguay; ^2^ Functional Genomics Laboratory, Institute Pasteur Montevideo, Montevideo, Uruguay; ^3^ Departamento de Parasitología y Micología, Facultad de Medicina, Universidad de la República, Montevideo, Uruguay; ^4^ Departmento Basico de Medicina, Facultad de Medicina, Hospital de Clinicas, Universidad de la República, Montevideo, Uruguay

**Keywords:** Argonautes (AGO), Toxoplasma, Trypanosoma, PIWI, piRNA biogenesis

## Abstract

piRNAs function as genome defense mechanisms against transposable elements insertions within germ line cells. Recent studies have unraveled that piRNA pathways are not limited to germ cells as initially reckoned, but are instead also found in non-gonadal somatic contexts. Moreover, these pathways have also been reported in bacteria, mollusks and arthropods, associated with safeguard of genomes against transposable elements, regulation of gene expression and with direct consequences in axon regeneration and memory formation. In this Perspective we draw attention to early branching parasitic protozoa, whose genome preservation is an essential function as in late eukaryotes. However, little is known about the defense mechanisms of these genomes. We and others have described the presence of putative PIWI-related machinery members in protozoan parasites. We have described the presence of a PIWI-like protein in *Trypanosoma cruzi*, bound to small non-coding RNAs (sRNAs) as cargo of secreted extracellular vesicles relevant in intercellular communication and host infection. Herein, we put forward the presence of members related to Argonaute pathways in both *Trypanosoma cruzi* and *Toxoplasma gondii*. The presence of PIWI-like machinery in Trypansomatids and Apicomplexa, respectively, could be evidence of an ancestral piRNA machinery that evolved to become more sophisticated and complex in multicellular eukaryotes. We propose a model in which ancient PIWI proteins were expressed broadly and had functions independent of germline maintenance. A better understanding of current and ancestral PIWI/piRNAs will be relevant to better understand key mechanisms of genome integrity conservation during cell cycle progression and modulation of host defense mechanisms by protozoan parasites.

## 1 Introduction

### 1.1 Introduction to piRNA biology

**FIGURE 1:**
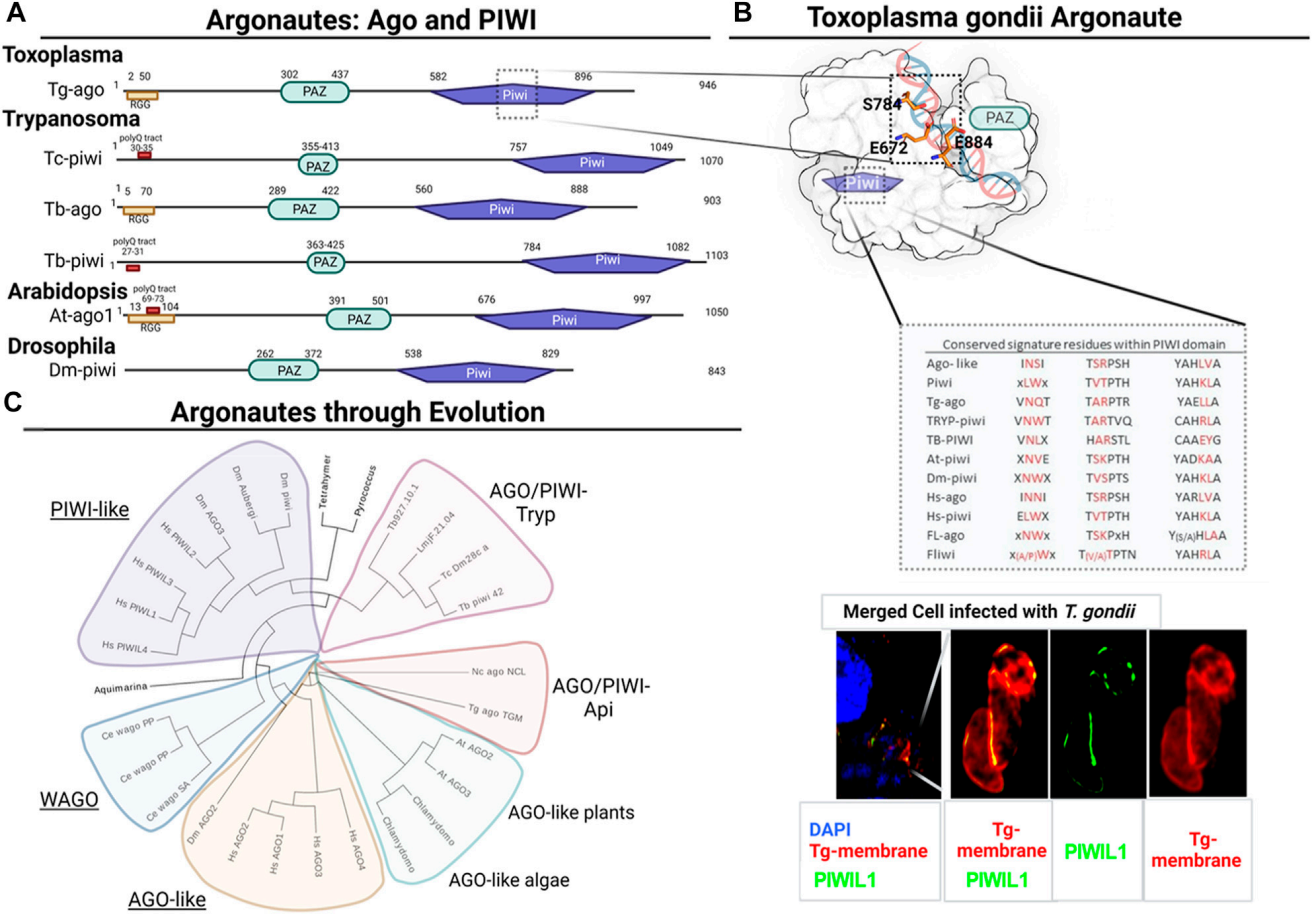
*Structural domain organization and phylogenetic analysis of Argonaute and PIWI proteins*. **(A)** Domain organization of PIWI proteins from different species. PAZ and PIWI are shown in cyan and purple boxes, respectively. RGG rich zone and poly Q tract are highlighted when present. Tg: *Toxoplasma gondii*, Tc: *Trypanosoma cruzi*, Tb: *Trypanosoma brucei*, At: *Arabidopsis thaliana*, Dm: *Drosophila melanogaster*. **(B)** Surface representation of Tg-Ago Δ-N-terminal structural model. Catalytic triad residues (ESE) are shown as orange sticks. Different motifs within Ago-proteins are compared with group of species, highlighting in red signature conserved residues that define type/class of Ago/piwi proteins. Immunofluorescence assay of Vero cells infected with TgRHΔku80 parasites was carried out using the Anti-TcPIWI Antibody (Garcia Silva et al., 2010a) (green), and Anit-SAG1 (red) was used to define parasites membrane. DAPI labels nuclei. Note that a single Z section is shown for clarity. **(C)** Phylogenetic relationships among Argonaute and Piwi proteins from the following species: At: *Arabidopsis thaliana*, Dm: *Drosophila melanogaster*, HS: *Homo sapiens*, Ce: *Caenorhabditis elegans*, Cr: *Chlamydomonas reinhardtii*, Lm: *Leishmania major*, Nc: *Neospora caninum*, Tb; *Trypanosoma brucei*, Tc: *Trypanosoma cruzi*, Tt: *Tetrahymena thermophila*, Tg: *Toxoplasma gondii*. Unrooted maximum likelihood tree of Argonaute proteins. Proteins among different species and clades were aligned using M-coffee (Wallace, 2006). Unrooted maximum likelihood tree with statistical branch support (SH-like) was generated with PhyML (Guindon et al., 2010) with smart model selection (SMS) (Lefort et al., 2017). Tree was visualized with Evolview (He et al., 2016).

The mobilization and regulation of expression of transposable elements (TE), genome stability and architecture maintenance, are essential cell functions of all organisms. Arguably the best-known TE expression silencing mechanism is RNA silencing (Fedoroff, 2012). Since its discovery, the study of RNAi pathways in early eukaryotes has drawn much interest. In the center of RNA silencing pathways are Argonaute proteins. Members of the Argonaute family are historically classified into two main classes: Argonaute-like (AGO) subfamily (from *Arabidopsis thaliana*) and PIWI-like subfamily (from *Drosophila melanogaster* PIWI) (Carmell et al., 2002). Argonaute proteins have three main domains: PAZ, MID and PIWI. The PAZ domain binds to the 3′end of guide RNAs. The 5′ phosphate of the small non-coding RNAs (sRNA; guide) binds to the MID domain. Then, cleavage takes place at the PIWI domain. Within its sequence, the PIWI domain contains a catalytic aspartate-aspartate-glutamate (D D E/D) triad. This triad works as an RNase H-like protein (Carmell et al., 2002) (Figure 1).

PIWI-interacting RNAs (piRNAs) are single-stranded RNA molecules that range between 21–35 nucleotides with 2′-O-methyl-modified 3′ ends (Ozata et al., 2019). The best characterized function of piRNAs is in genome defense against TE insertion in germ line cells.

Akin to microRNAs (miRNAs), the main function of piRNAs is performed by Argonaute proteins of the PIWI subfamily clade (Aravin et al., 2006). Generally, piRNAs bind to PIWI proteins to target transcripts coming from piRNA clusters, TE regions or mRNAs *via* base-pair complementarity, inducing their endonucleolytic cleavage and generating, in turn, new piRNAs (reviewed in Yamashiro and Siomi, 2018).

However, the biogenesis of piRNAs is complex and varies amongst organisms, making it difficult to draw a general diagram. In flies and mammals, mature TE-derived piRNAs are produced and processed from heterochromatic regions of the genome called piRNA clusters. These are long genomic stretches that produce several piRNAs (Brennecke et al., 2007) (recently reviewed in Haase, 2022). In *Drosophila* and mice, piRNAs can be produced from 3′ UTRs and protein coding regions of several mRNAs (Gainetdinov et al., 2018).

**FIGURE 2:**
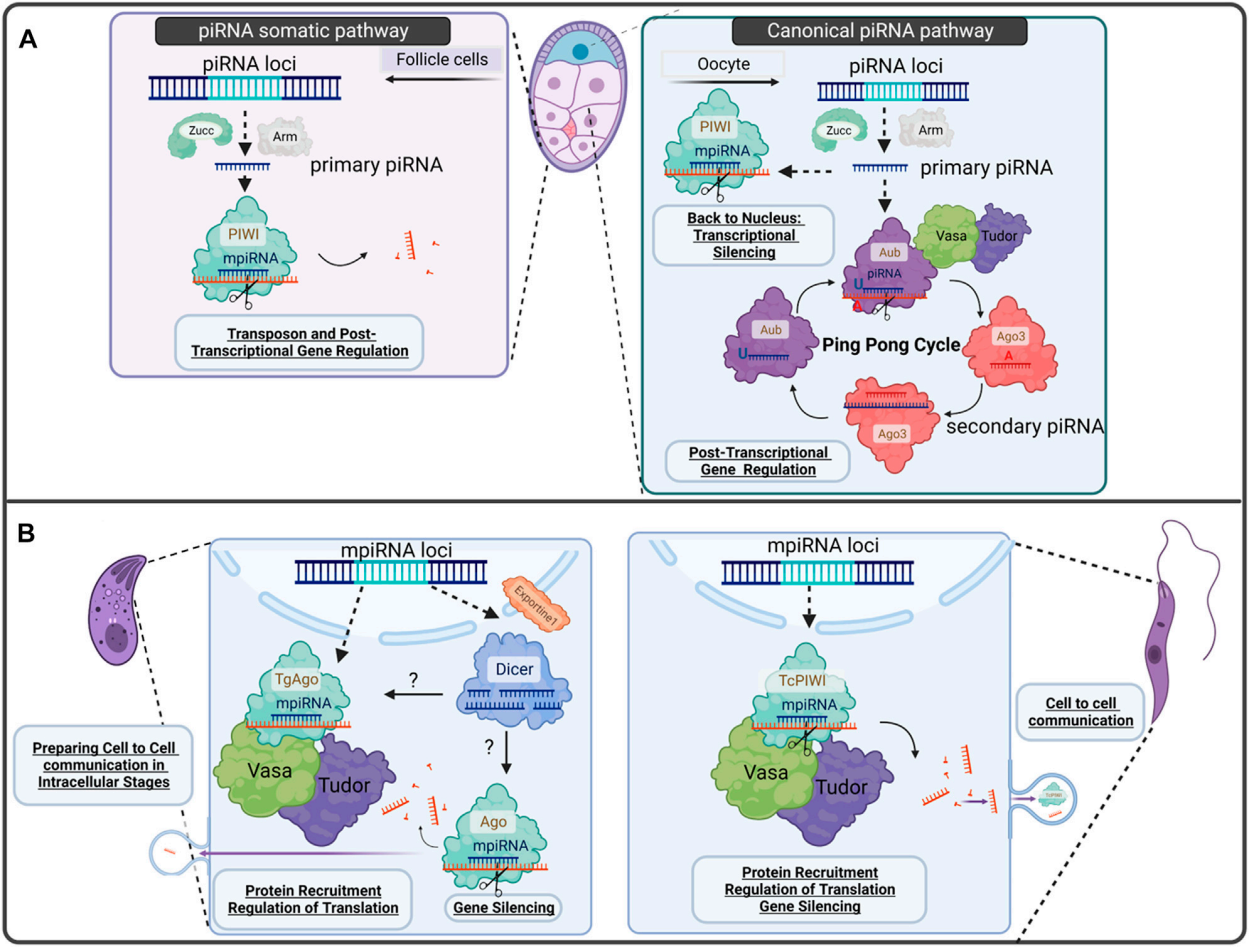
*Gonadal-piRNA pathway* vs. *proposed model for Protozoa parasites -related piRNA pathways discussed in this perspective*. **(A)** *Canonical piRNA pathway found in D. melanogaster egg chamber*. On the left, we can observe the somatic piRNAs at follicle cells where transcribed piRNA cluster by RNA Pol II RNA polymerase are processed in primary piRNAs, binds to PIWI proteins and are guided to silence TE invaders or target mRNAs for post-transcriptional gene regulation. On the right, the canonical piRNA pathway in germline cells from the ovary of *D. melanogaster* is shown. Primary piRNA biogenesis occurs in follicle cells at the *nuage*. There, there is a piRNA amplification process called *ping-pong cycle.* The piRNAs produced during this process are called *secondary piRNAs*). Primary piRNAs can also bind to PIWI protein and go back to the nucleus to remodel chromatin, for example, and regulate gene transcription. **(B)** Proposed model for Apicomplexa and Trypanosomatids PIWI-like proteins discussed in this perspective. *T. gondii* expresses TgAgo, Vasa, Tudor and Dicer. We proposed an intermediate mechanism between RNAi and piRNA biogenesis, where mpiRNAs are transcribed in the nucleus to reach the cytoplasm to find Dicer or Ago/PIWI-like proteins. After being processed, mpiRNAs, they can either be secreted to mediate cell to cell, and cell-host communication or target mRNAs to control gene expression. In *T. cruzi*, mpiRNAs are composed mainly of tRNA halves recruited by TcPIWI-tryp and in *T. gondii* TgAgo: srRNAs and miRNAs.

In *Drosophila melanogaster*, for example, secondary or responder piRNA production and amplification are the result of placing primary (initiator) piRNAs into the so called “*ping-pong*” cycle. This entails the cleavage of piRNA cluster transcripts to generate intermediates which further mature. Resulting piRNAs (responder piRNAs) are 24–30 nucleotides (nt) long, with a bias for a 5′uracil (5′U) and perfect complementarity for their targets. Tudor nuclease (localized between the *nuage* and mitochondria) tether PIWI proteins to the mitochondrial outer membrane and start primary piRNA processing. A second mechanism responsible of generating responder piRNAs, is initiated at the outer mitochondrial membrane by the endonuclease Zucchini (Zuc) or PDL6, in *D. melanogaster* or mammals, respectively, associated with several proteins (Ozata et al., 2019). Primary transcribed piRNAs are then converted to tail-to-head trailed/phased piRNAs (Ozata et al., 2019). In the best characterized systems, the *ping-pong cycle* and processing from phasing piRNA mechanisms generate the main complexity of piRNA population (Gainetdinov et al., 2018) (Figure 2. Panel A).

The components of piRNA pathways are localized to different subcellular regions depending on their function. For example proteins that participate in piRNA biogenesis -including Vasa (a DDX-4 homolog of mammals) and Armitage, among others-, are localized near the *nuage* in fly germ cells or Yb bodies in the surrounding somatic follicular cells (Rogers et al., 2017).

Another well studied model in piRNA biology is *Caenorhabditis elegans*. In *C. elegans*, two main populations of piRNAs not linked to *ping-pong cycles* are found: 21U and 22G (Hoogstrate et al., 2014). These are 21 and 22 nt long, have 5′U/G bias respectively, and do not match perfectly onto their targets. Instead, nematode piRNAs are produced by PIWI-like proteins named PRG-1 and WAGO-9, and are efficiently maintained by an RNA-dependent RNA polymerase (RdRP) (Suleiman et al., 2022). Overall, these differences among models may reflect the diversity of PIWI/piRNAs machinery components.

### 1.2 Misidentification of piRNAs: Canonical vs. miscellaneous piRNAs

Although the role of piRNAs in germline appears to be conserved among animals, piRNAs have also been reported to have germline-independent functions (somatic piRNAs). Some examples are found in multicellular organisms, such as *Aedes aegypti*, where somatic piRNAs act against viruses. piRNAs also silence transposons in the *D. melanogaster* body fat and brain tissues. Furthermore, the mollusk *Aplesia californica* presents a central nervous system memory function where piRNAs are involved (Lewis et al., 2018). Overall, somatic piRNAs seem to be ubiquitous across organisms, where they target TEs and messenger RNAs. However, there are conflicting reports regarding the expression of canonical piRNAs and the relevance of PIWI in somatic tissues, outside of the Protista kingdom. In regards to this, it has been recently reported that a large part of the so-called somatic piRNAs, which have been described in non-germ tissues, derive from non-coding RNAs, which do not bear the specific molecular properties of canonical piRNAs (Tosar et al., 2018).

In order to clarify misidentifications due to piRNA nomenclature, we have introduced previously, the concept of miscellaneous-piRNAs (mpiRNAs). The term mpiRNAs aims to distinguish between canonical piRNAs and other sRNAs that are circumstantially associated with PIWI proteins in somatic contexts (Tosar et al., 2021). The mpiRNAs would be RNAs that, without being within canonical “piRNA clusters” at the genomic level (and therefore, not corresponding to the classification of piRNAs), could be associated with PIWI proteins in certain contexts, mediating their interaction with other proteins or, more likely, with other transcripts or even with the genome. As an example, it has been described that nematodes outside of Clade V use an alternative class of sRNAs to compensate for the loss of piRNAs to regulate TE activity (Suleiman et al., 2022). Therefore, it would be interesting to analyze whether the pathway has disappeared over time, from early to modern eukaryotes (Metazoans).

### 1.3 Argonaute-related pathways in evolution: Revisited?

Dicer, an RNase III endonuclease, Argonaute and PIWI proteins have been found across all domains of life. Phylogenetic studies supported the idea that the Last Eukaryotic Common Ancestor (LECA) used the RNAi pathway, which uses sRNAs as a guide, to control genome invasions (Cerutti and Casas-Mollano, 2006). Likewise, Argonaute family orthologues have been identified in both archaeal and bacterial super-kingdoms, and were named prokaryotic PIWI (pPIWI) (Burroughs et al., 2013). The comparative roles of pPIWI proteins vs. eukaryotic PIWIs (ePIWI) have remained scarcely studied. Furthermore, some authors have described the presence of divergent PIWI subfamilies in prokaryotes: PIWI-RE. PIWI-RE display conserved arginine (R) and glutamic acid (E) residues, and are predicted to function as other members of the PIWI family, as part of an RNA-dependent restriction system to target DNA from phages, plasmids or conjugative transposons (Burroughs et al., 2013; Swarts et al., 2014). Interestingly, a recent study shows that PIWI-RE exhibits unique endonuclease activity, including RNA-guided DNA cleavage and DNA-guided RNA cleavage and have a relevant role in bacterial cell division (Huang et al., 2022).

The origin of piRNAs remains controversial. Similar to the recently challenged common idea that miRNAs originated in the Animal kingdom (Fridrich et al., 2020), piRNAs could also have arisen earlier on the tree of life. While piRNAs seem to be restricted to animals and seemingly just present in the Animal kingdom, PIWI proteins, can be found, for example, in ciliated single celled eukaryotes, such as *Tetrahymena thermophila* (Mochizuki and Gorovsky, 2004) and *Paramecium tetraurelia* (Singh et al., 2014). Interestingly, *Tetrahymena* requires PIWI proteins to accomplish its sexual life cycle, as *D. melanogaster* requires an active piRNA pathway to complete normal embryo development (Juliano et al., 2011).

Herein, we highlight the expression of Argonaute and piRNA pathway-related proteins in two protozoan parasites with relevance in human health: *Trypanosoma cruzi* and *Toxoplasma gondii*.

### 1.4 piRNA pathway in protozoan parasites: Trypanosoma and Apicomplexa examples

RNAi machinery can be traced back to the LECA (Cerutti and Casas-Mollano, 2006). However, this pathway appears to be simplified or absent in unicellular organisms such as *Trypanosoma cruzi* or *Leishmania major*. For other organisms of the same family, however, the RNAi machinery is fully functional, such as is the case of *Trypanosoma brucei* (Ullu et al., 2004). We have performed both experimental and phylogenetic analyses of the sRNA-related machinery components in some of these model organisms. Surprisingly at the time, we reported the expression of an ancestral PIWI ortholog of AGO/PIWI in *T. cruzi*, which led us to propose the generic designation of PIWI-tryp (Garcia Silva et al., 2010a). Subsequently, we also reported the presence of sRNAs that derived from tRNAs, which we later named tRNA-fragments (tRFs). These were bound to PIWI proteins from Trypanosomatids (Garcia-Silva et al., 2010b). We also reported that these molecules were secreted as part of EV’s cargo to the extracellular space. The secretion of these protein is key in pathogenesis and intercellular communication (Garcia-Silva et al., 2014; Retana Moreira et al., 2019). Our work along with that of others, concluded that the evolution of the PIWI subfamily of proteins is highly conserved, from prokaryotes to modern eukaryotes. This entails that, in their ancestral state, PIWI proteins could have been expressed broadly and had functions independent of germline maintenance (Ross et al., 2014). In line with this, recent reports suggest the existence of an active system of non-coding RNAs (Matrajt, 2010; Wang et al., 2012; Li et al., 2020; Jung et al., 2022; Peng et al., 2022; Zhang et al., 2022), including miRNAs (Rojas-Pirela et a.l, 2022) in the Apicomplexa phylum. Furthermore, EVs secreted from *Toxoplasma* have recently been reported and characterized (Wowk et al., 2017; Silva et al., 2018).

The Apicomplexa phylum is exclusively composed of parasitic protists. Among others, the phylum encompasses species of *Plasmodium,* and *Toxoplasma gondii,* the agents of malaria and toxoplasmosis, respectively. Similar to Trypanosomatids, some organisms from the Apicomplexa family do not present RNAi or Argonaute orthologs. That is the case of *P. falciparum* where dsRNAs are able to trigger gene silencing (Riyahi et al., 2006). *Toxoplasma* has a complex life cycle with different stages, most of which are haploid. Sexual forms (macro- and microgametes) are only present within the guts of Felid species (their definitive host). Sexual recombination takes place in order to generate a diploid oocyst, which upon sporulation gives rise to eight sporozoites.

Despite there being no evidence for Toxoplasma-PIWI-like proteins, Braun and others characterized TgAgo, an Argonaute homolog, responsible for RNAi-type mechanisms in *T. gondii* (Braun et al., 2010). Non-etheless, TgAgo’s slicer activity is only detected in the presence of Mg^2+^ and upon a perfect antisense RNA guide match (Musiyenko et al., 2012). TgAgo presents the four characteristic domains of Argonaute proteins: N- terminal, PAZ, Mid and a C-terminal PIWI domain (Hutvagner and Simard, 2008) (Figure 1A). The N-terminal domain is highly unstructured and contains RGG rich tracts similar to the ones characterized in *Trypanosoma*, *Leishmania* and *Arabidopsis* (Shi et al., 2004a; Shi et al., 2004b; Lye et al., 2010). This region usually has essential roles in the metabolism of miRNAs (Rajyaguru and Parker, 2012) and presents arginines that undergo methylation. RGG motifs were demonstrated to bind eIF4G and repress mRNA translation (Rajyaguru and Parker, 2012), TgAgo was shown to bind yet another RGG motif containing protein, TgAlba (Braun et al., 2010). TgAlba is involved in translational control of gene expression particularly associated with stress response and differentiation (Gissot et al., 2013; Boulila et al., 2014). TgAgo can also bind to Tudor domain-containing proteins, which are subcellularly localized to cytoplasmic granular structures. Moreover, TgAgo was observed to be associated to polysome fractions (Musiyenko et al., 2012).

Additionally, Braun et al argue that TgAgo resembles more a PIWI-like or piRNA-related protein. If we consider model organisms, such as *D. melanogaster* or mice, PIWI proteins are associated with canonical and non-canonical piRNAs pathways (Ramat et al., 2020). Interestingly, proteomic data [available on toxoDB.org Release 61 (Harb and Roos, 2020)] reveals the expression of TgAgo in *Toxoplasma* oocysts (Fritz et al., 2012), the product of sexual replication. This raises the possibility that TgAgo might share germline-associated functions similar to those reported for metazoan PIWI in the context of oocysts.

Although TE are windspeed in eukaryotes, Apicomplexan are the best-known exception. *Plasmodium falciparum*, *Toxoplasma gondii*, *Encephalitozoon intestinalis* and *Theileria parva*, seem to have purged TEs from their genomes (Wells and Feschotte, 2020). Instead, REP elements (mitochondrial-like sequences organized as direct or inverted repeats) are dispersed throughout the nuclear genome of Toxoplasma (Ossorio et al., 1991). REP sRNAs (rdsRNAs) are bigger than typical miRNA or siRNAs (21–27 nt). About half of the rdsRNAs have a 5′ terminal U, like responder piRNAs. Some of the most abundant sRNAs expressed in *T. gondii*, besides miRNA-like molecules, match to REP elements and repeat-associated sRNAs. Repeat-associated sRNAs map perfectly onto high-copy-number satellite DNA Sat350 in the genome of *T. gondii* (Braun et al., 2010). Therefore, it was previously proposed that TgAgo might contribute to post-transcriptional gene silencing of repeats and transposons *via* Tg-dsRNAs, as is seen with metazoan PIWI proteins (Musiyenko et al., 2012)

The resemblance of PIWI-tryp and TgAgo to the PIWI subfamily led us to revisit phylogenetic, structural and sequence alignment analyses in selected organisms to update our understanding of the matter.

We looked for specific members related to AGO/PIWI proteins in Protists, Algae, Plants, Metazoan and Prokaryotes in order to compare sequence alignments for the presence or absence of different domains (Figure 1).

We further performed sequence homology analyses to determine the presence of conserved members of the piRNA pathways in *Toxoplasma gondii*. We found homology with proteins such as Dicer (Braun et al., 2010), VASA, Tudor and several others related to RNA metabolism.

TgAgo forms complexes with many proteins which have orthologues in human and *Drosophila* RISCs (Braun et al., 2010; Musiyenko et al., 2012). It has been demonstrated that, in addition to binding to a stronger RNA slicer, a Tudor staphylococcal nuclease (TSN), TgAgo can bind to PRMT1 (Braun et al., 2010). PRMT1, belongs to the family of arginine methyltransferases that use RGG motifs as substrates. Interestingly, the subcellular localization of PRMT1 is pericentrosomal. PRMT1 knockout parasites show defects in cell division and in the number of centrosomes (El Bissati et al., 2016). This might suggest an association between PRMT1 and RISC with cell cycle regulation. Using an antibody specific to *T. cruzi* PIWI protein, we detect in *T. gondii* labeling of cytoplasmic structures reminiscent of PRMT1’s localization. Whether this labeling corresponds to the localization of TgAGO needs further exploration (Figure 1).

Usually the PIWI domain presents an RNAse H-like tertiary structure, with a more relaxed catalytic center (Hutvagner and Simard, 2008). In *Toxoplasma*, as well as in *Neospora caninum*, *Cystoisispora* sp. and *Hammondia sp*, the catalytic residues are E/S/E (Figure 1B), presenting variations compared to the conserved D/D/H triad, responsible for the nuclease activity, which in turn could explain its previously reported relative weak RNA cleavage activity (Musiyenko et al., 2012). Furthermore, TgAgo, as well as its homologs in all the Apicomplexa we analyzed, share the same sequence signatures. These signatures are particularly different from the ones characterized for PIWI-like and Ago-like proteins. Non-etheless, TgAgo does not cluster with those of other protozoa (Figure 1C). The branch where it clusters is phylogenetically related to AGO-like proteins from plants and algae, as it was previously described (Braun et al., 2010).

Further studies on Argonaute proteins from other members of the Apicomplexa clade would be required to functionally classify them as PIWI-like, Ago-like, or under the recently defined WAGO proteins. Herein, we put forward PIWI-Api, out of analogy to Piwi-Tryp, to denominate this specific cluster. Understanding the evolution and relevance of PIWI proteins and pathways in Apicomplexan, could have implication for targeted drug discovery as they might relate to the ones of plants and be involved in parasite division or differentiation.

### 1.5 piRNA pathway before animals

As we noted before, PIWI family of proteins is better known for their ancestral genome defense functions which later evolved into gene expression regulation functions in modern eukaryotes. It is possible that the PIWI protein already had a genome defense function at the early eukaryotic level. Alternatively, PIWI could have evolved from genomic defense to a gene expression regulation with the passing of time. In trying to elucidate these alternatives, authors have proposed that transposons could be regarded as genomic pathogens causing genomic instability. In this context, piRNAs surge as defense mechanisms against transposons at the genome level. As a result, piRNA machineries are fast evolving as a consequence of the so-called “Red Queen arms race” between transposons and the piRNAs pathway. This in turn, contributes to genome evolution and generates genetic diversity, increasing the system’s complexity (Parhad and Theurkauf, 2019). All these, led to the concept of piRNAs as regulators of gene expression beyond the biology of transposons in somatic tissues, as exemplified here for *T. gondii* and *T. cruzi*. Interestingly, in *Hydra*, somatic expression of PIWI proteins is well documented and has been linked to the ability of asexual reproduction (Lim et al., 2014). Our previous reports on these organisms show that the gene expression regulation and the regulation of TE mediated by the PIWI protein were already an ancestral characteristic of the protein uncovering an early evolutionary origin for somatic functions of the piRNA pathway.

## 2 Discussion

Within the germline of animals, both PIWI and piRNAs are highly abundant, ensuring genome integrity of germ cells. We propose here that PIWI machinery could represent an evolutionary conserved pathway, consequence of an arms race between genomes against invading pathogens and the hosts, demonstrated by its expression in early branches of evolution (Figure 2A).

In addition, non-gonadal expressed PIWI proteins recruit certain cellular transcripts in the absence of canonical piRNAs. We pose here several unanswered questions in this field. In a scenario with no canonical piRNA pathways, would the ancestral Argonaute protein genes be defending the genome against a TE invasion? For the same group of organisms, the pathways are extremely diverse. From it being absent in the organism to being complex, with more than one protein acting as a key player.

We analyzed here two examples of vastly studied protozoan models from Apicomplexa and Trypanosomatid groups with implications for human and animal health. Although far related, they share complex life cycles that alternate between different stages of intracellular and extracellular forms. Besides, they have intermediate and definitive hosts, and treatments are either not effective or there exists resistance to drugs. In summary, it would be relevant to find molecular targets in these parasites with no homology when comparing to their hosts. We then propose the Argonaute family of proteins as potential specific candidates to be explored in the quest for new therapies to control parasitic infections.

Ongoing research aims to better understand the evolutionary origins of RNAi pathways, which will lead to better understanding of their functions. Non-etheless, PIWI-piRNAs pathways remain a puzzling group of sRNAs with emerging novel mechanisms and functions. Why were Argonaute proteins lost in some lineages? What would be the driving force behind the specialization process of the PIWI family of proteins? Future research tackling these open questions will shed light onto these ill-understood aspects of RNAi pathways of gene expression regulation.

## Data Availability

The original contributions presented in the study are included in the article/supplementary material, further inquiries can be directed to the corresponding author.
